# Differential detection of nuclear envelope autoantibodies in primary biliary cirrhosis using routine and alternative methods

**DOI:** 10.1186/1471-230X-10-28

**Published:** 2010-03-08

**Authors:** Elena Tsangaridou, Hara Polioudaki, Rania Sfakianaki, Martina Samiotaki, Maria Tzardi, Meri Koulentaki, George Panayotou, Elias Kouroumalis, Elias Castanas, Panayiotis A Theodoropoulos

**Affiliations:** 1 Department of Biochemistry, School of Medicine, University of Crete, Crete, Greece; 2 Department of Gastroenterology, School of Medicine, University of Crete, Crete, Greece; 3 Biomedical Sciences Research Center "Alexander Fleming", Vari 16672, Greece; 4 Department of Pathology, School of Medicine, University of Crete, Crete, Greece; 5 Department of Gastroenterology, University Hospital of Heraklion, Heraklion, Crete, Greece; 6 Department of Experimental Endocrinology, School of Medicine, University of Crete, Crete, Greece

## Abstract

**Background:**

Detection of autoantibodies giving nuclear rim pattern by immunofluorescence (anti-nuclear envelope antibodies - ANEA) in sera from patients with primary biliary cirrhosis (PBC) is a useful tool for the diagnosis and prognosis of the disease. Differences in the prevalence of ANEA in PBC sera so far reported have been attributed to the methodology used for the detection as well as to ethnic/geographical variations. Therefore, we evaluated the prevalence of ANEA in sera of Greek patients with PBC by using methods widely used by clinical laboratories and a combination of techniques and materials.

**Methods:**

We screened 103 sera by immunoblotting on nuclear envelopes and indirect immunofluorescence (IIF) using cells and purified nuclei. Reactivities against specific autoantigens were assessed using purified proteins, ELISA, immunoprecipitation and mass spectrometry.

**Results:**

We found higher prevalence of ANEA when sera were assayed by IIF on purified nuclei or cultured cells (50%) compared to Hep2 commercially available slides (15%). Anti-gp210 antibodies were identified in 22.3% and 33% of sera using ELISA for the C-terminal of gp210 or both ELISA and immunoprecipitation, respectively. Immunoblotting on nuclear envelopes revealed that immunoreactivity for the 210 kDa zone is related to anti-gp210 antibodies (p < 0.0001). Moreover, we found that sera had antibodies for lamins A (6.8%), B (1%) and C (1%) and LBR (8.7%), whereas none at all had detectable anti-p62 antibodies.

**Conclusions:**

The prevalence of ANEA or anti-gp210 antibodies is under-estimated in PBC sera which are analyzed by conventional commercially available IIF or ELISA, respectively. Therefore, new substrates for IIF and ELISA should be included by clinical laboratories in the analysis of ANEA in autoimmune sera.

## Background

Nuclear envelope is a complex structure consisting of outer and inner nuclear membranes, nuclear pore complexes (NPCs) and the nuclear lamina [1]. The outer nuclear membrane represents an extension of the endoplasmic reticulum, whereas the inner part constitutes a specialized environment that accommodates a unique set of proteins (LBR, emerin, LAP1s, and LAP2s). The nuclear lamina is composed of A- and B-type lamins. These proteins form a polymeric lining that supports the inner nuclear membrane and imparts elasticity to the nuclear envelope. NPCs provide the sole means for regulated transport between the cytoplasm and the nucleoplasm and are conserved in all eukaryotic cells, from yeast to human. The mammalian NPCs are 125-MDa complexes containing 30 distinct polypeptides, called nucleoporins [2].

In a number of diseases, such as autoimmune liver and systemic rheumatic pathologies, a correlation with autoantibodies against nuclear envelope (ANEA) was reported [3]. Among them, primary billiary cirrhosis (PBC) is one of those in which ANEA have been considered as pathognomonic element [4,5]. However, a significant variation of their prevalence (between 10% and 48%) has been reported, when indirect immunofluorescence (IIF) is used for the screening of PBC sera [4,6-11]. This could be attributed to differences on the processing of IIF samples, namely substrates and reagents used for the detection and on the evaluation of the results, especially when antibodies of cytoplasmic specificities are present in the same serum [3,4,11].

PBC sera may contain a number of autoantibodies against specific constituents of the nuclear envelope. Antibodies against proteins of the nuclear pore complex, such as gp210, an integral glycoprotein of the nuclear pore membrane, and p62, a nucleoporin of the central channel, have been reported [10,12], being associated with the activity and severity of PBC [13]. In addition, it was recently suggested that anti-gp210 antibodies may be related to the hepatic failure-type of the disease [14]. The presence of anti-gp210 autoantibodies in PBC sera has been reported for the first time in 1990 [15] and shortly after, a 15-amino acid linear stretch within the carboxy-terminal domain of the protein, has been shown to be the predominant epitope [16]. Moreover, autoantibodies against gp210 have been demonstrated to recognize at least two different epitopes: one within the cytoplasmic tail and another located within the large glycosylated lumenal domain [17]. However, thereafter and until today, anti-gp210 antibodies in sera of patients with PBC from USA [18], Europe [9,19-22] and Asia [14,23,24] were identified essentially by ELISA, using as an antigen the carboxy-terminal domain of the protein. These studies have shown different prevalence (10.4%-44%) for anti-gp210 autoantibodies, which have been essentially attributed to geographical and/or ethnic variations.

Autoantibodies against p62 have been reported for the first time in 1996 by two groups in Europe and Japan [8,10]. Using immunoblotting, they have shown that antibodies in PBC sera recognize a 62 kDa protein in a nuclear pore complex-enriched preparation with a prevalence of 32% in PBC patients [10]. An immunoreactive 62 kDa band was shown with a similar frequency (31%), after immunoblotting of PBC sera on a WGA-bound fraction of rat liver nuclear envelopes [7]. Using recombinant autoantigens, PBC sera was found to react more frequently with p62 (55%) than gp210 (10%) nucleoporin [25] and that more than 50% of PBC sera precipitated ^35^S-radioactively labeled p62 recombinant rat or human nucleoporin, while 40% recognized this recombinant antigen by immunoblotting [26].

Antibodies against lamin B receptor (LBR), an integral protein of the inner nuclear membrane, are specific for PBC and when detected in sera, their positivity ranged from 1% to 9% [7,9,15]. It has been also shown that anti-LBR autoantibodies recognize intact or the N-terminal domain of human, rat and chicken LBR [15,27,28].

The aim of this study was to establish an IIF procedure, which allows the efficient and easy detection of ANEA and to determine the prevalence of different ANEA autoantibody classes, specific for PBC. We report that ANEA and more specifically anti-gp210 are present with high frequency in PBC sera. Antibodies against other components of the nuclear envelope, such as LBR and lamins, are detected with low prevalence, whereas anti-p62 antibodies are absent.

## Methods

### Patients and sera

Serum samples from one hundred and three (103) patients (92 female and 11 male) with established PBC were used. All patients were followed at the Department of Gastroenterology, University Hospital of Heraklion, Greece. PBC was diagnosed according to established clinical, laboratory and histological findings [29] and 86% of sera contained anti-mitochondrial antibodies. In addition, we analyzed by IIF sera from 10 healthy controls, 25 individuals without autoimmune diseases registered with the university hospital, 5 patients with viral hepatitis B, 5 patients with viral hepatitis C and 10 patients with alcoholic cirrhosis. All patients consented to be included in the study which has been approved by the Ethics and Scientific Committees of the University Hospital of Heraklion.

### Antibodies

Mouse monoclonal antibody p62 (BD Transduction Laboratories, Becton Drive, Franklin Lakes, New Jersey, USA) or mouse monoclonal antibody 414 which binds to FXFG-containing nup358, nup214, nup153, p62 and nup54 (Covance, Emeryville, California, USA) were used for immunoprecipitation and immunoblotting experiments. The characterization of affinity purified, rabbit polyclonal anti-chicken LBR has been described previously [30].

### Cell lines and culture

HeLa cells (cervical adenocarcinoma) were cultured in Dulbecco's MEM (Biochrom, Berlin, Germany), supplemented with 10% heat-inactivated fetal bovine serum, penicillin and streptomycin. HepG2 cells (Human Hepatocellular liver carcinoma cell line) were maintained in DMEM/HAM'S F-12 (1:1) with N- Acetyl - L- Alanyl- L- glutamine (Biochrom, Berlin, Germany) containing 10% FBS, penicillin, and streptomycin. Both cell lines were obtained from the American Type Tissue Culture Collection (Manassas, VA) and were grown at 37°C in a humidified incubator with a 5% CO_2 _atmosphere.

### Isolation of nuclei from rat liver and HepG2 cells

Rat liver nuclei were prepared as described by Blobel and Potter [31]. To isolate nuclei from HepG2 cell cultures, single cells were incubated in ice-cold hypotonic buffer and then Dounce-homogenized as described [11]. Purity of nuclei was examined by indirect immunofluorescence and immunoblotting.

### Immunostaining and Confocal Microscopy

Sera (dilution 1/80) were tested on HeLa and HepG2 cells grown on coverslips and isolated nuclei from rat liver and HepG2 cells immobilized on coverslips using alcian blue by indirect immunofluorescence (IIF) performed as described previously [11]. Sera (dilution 1/80) were also analyzed using commercially available Hep2 cells (Inova Diagnostics, San Diego, CA) following the instructions of the supplier. In all cases, detection was made by a supplied FITC-coupled anti-human IgG antibody, diluted and ready for use (Inova Diagnostics, San Diego, CA) or FITC-coupled anti-human antibody IgM (DAKO, Carpinteria, CA) at a dilution of 1/100. Fluorescence was routinely assayed in a Leica SP confocal microscope (Leica microsystem Heidelberg GmbH, Germany)

### Preparation of nuclear envelopes and proteins of the nuclear envelope

Nuclear envelopes were prepared from isolated rat liver nuclei following DNAse and RNAse digestion and washes with high salt buffer and ice-cold distilled water as previously reported [11].

Lamins A, C and lamin B were extracted from rat liver nuclear envelopes (RNE) with 8 M urea and further purified by ion exchange chromatography (DE52, Whatman International Ltd Maidstone, UK). The nucleoplasmic amino-terminal domain of chicken LBR fused to glutathione-S-transferase (GST) was expressed in BL21 (DE3) cells and purified from lysates according to standard procedures.

### Immunoblotting

Purified proteins, RNE, or immunoprecipitated material were electrophoresed on a 7.5% SDS polyacrylamide gel, transferred on nitrocellulose membrane and immumoblotted as previously described [11]. PBC sera, anti-p62 and anti-LBR antibodies were used at 1/300, 1/1000 and 1/200 dilution, respectively. Autoantibodies were recognized using anti-human Ig, IgG (GE Healthcare, Buckinghamshire, UK), (1/10000) or IgM, (Southern Biotech, Birmingham, Alabama, USA) (1/8000) secondary antibodies; monoclonal or polyclonal antibodies were detected using anti-mouse or anti-rabbit secondary antibodies, all conjugated with HRP (GE Healthcare, Buckinghamshire, UK) at a dilution of 1/10000 and detected using the ECL system (Thermo Scientific, Rockford, USA).

### Immunoprecipitation

Immunoprecipitation of nucleoporin p62 was accomplished using monoclonal antibodies and extracts from RNE. Nuclear pore proteins were extracted from RNE with buffer S1 (10% sucrose, 40 mM Tris-HCl pH 7.5, 300 mM NaCl,, 1 mM EGTA, 2 mM MgCl_2_, 1 mM PMSF, protease inhibitors) containing 0.3% Empigen BB (Fluka, St Louis MO, USA) [32]. Extract was first clarified by centrifugation (15000 g for 15 min, at 4°C) and then cleared from proteins which bound to sepharose beads by incubating clarified supernatant for 1 hour, at 4°C with protein G-Sepharose beads (GE Healthcare, Buckinghamshire, UK), already treated with 1% filtered fish skin gelatine. Cleared extract was incubated with 4 μg of mouse monoclonal anti-p62 antibodies (BD Transduction Laboratories, Becton Drive, Franklin Lakes, New Jersey, USA) or mouse monoclonal antibodies 414 (Covance, Emeryville, California, USA), overnight at 4°C and then with gelatine treated protein G-Sepharose beads, for 1.5 hour at 4°C. The immune complexes were recovered by centrifugation, washed 7 times with buffer S1 and solubilized in loading buffer by heating at 100°C.

For the immunoprecipitation of nuclear envelope antigens, autoantibodies were either absorbed or covalently coupled to protein G-Sepharose beads (GE Healthcare, Buckinghamshire, UK). In both protocols RNE were extracted with buffer S2 (2% Triton X-100, 20 mM Tris-HCl pH 7.5, 300 mM NaCl, 10% sucrose, 1 mM EGTA, 2 mM MgCl_2_, 1 mM PMSF, protease inhibitors) for 1 hour on ice. Extract was clarified by centrifugation at 15000 g for 15 minutes at 4°C and mixed with an equal amount of buffer without NaCl and Triton X-100. Finally, extract was incubated with gelatine treated protein G-Sepharose beads, for 1 hour, at 4°C, in order to be cleared from proteins which they bound to sepharose beads.

In the first protocol, sera were incubated with extract overnight at 4°C. The immune complexes were then incubated with protein G-Sepharose beads for 1.5 hour at 4°C, washed 7 times with buffer (1% Triton, 150 mM NaCl, 20 mM Tris-HCl pH 7.5, 10% sucrose, 1 mM EGTA, 2 mM MgCl_2_, 1 mM PMSF, protease inhibitors) and eluted in loading buffer by heating at 100°C for 15 minutes.

Alternatively, autoantibodies were covalently coupled to protein G-Sepharose beads essentially as previously reported [33]. For this, sera were incubated with protein G-Sepharose beads for 1 hour at 4°C, washed twice with 0.1 M sodium tetraborate, pH 9 and then incubated twice with freshly prepared 20 mM DMP (Dimethyl pimelimidate dihydrochloride, Sigma, St Louis MO, USA) in 0.1 M sodium tetraborate, pH 9 for 30 minutes at room temperature. Beads were washed two times with ice-cold 50 mM glycine, pH 2.5 to remove uncoupled autoantibodies, and then 7 times with PBS. Beads bearing the covalently linked autoantibodies were incubated with extract overnight at 4°C. The immune complexes were recovered by centrifugation and washed 7 times with buffer (1% Triton, 150 mM NaCl, 20 mM Tris-HCl pH 7.5, 10% sucrose, 1 mM EGTA, 2 mM MgCl_2_, 1 mM PMSF, protease inhibitors). The elution of bound autoantigens was performed by adding to beads an equal volume of loading buffer and heating at 70°C for 15 minutes.

### Mass spectrometry

Stained protein bands were in-gel digested with trypsin. The extracted peptides were analysed by mass spectrometry using an LCQ-Deca mass-spectrometer as previously described [34] while protein identification was performed with TurboSEQUEST software.

### Enzyme-linked immunosorbent assay

The antibody titers to gp210 were determined using ELISA kit (Inova Diagnostics, San Diego, CA) according to the manufacturer's protocol and instructions.

### Statistical analysis

Statistical analysis was performed with the SPSS V16 (SPSS, Chicago, IL) program. Comparison between values was accomplished using the χ^2 ^test.

## Results

### Routine histological slides do not detect ANEA in all positive sera of PBC patients. Alternative substrate-fixation methods

**Figure 1:**
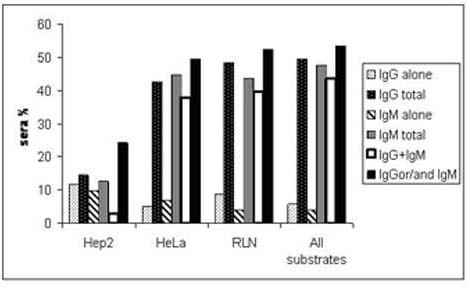
**Prevalence of ANEA**. Histograms showing the percentage of PBC sera containing ANEA of different Ig class as determined by IIF on Hep2 slides, HeLa cells, rat liver nuclei (RLN) and either substrate. "IgG Total" or "IgM Total" represent the sum of sera which had ANEA of both IgG and IgM antibodies (IgG+IgM) and those with either IgG class alone or IgM class alone, respectively.

**Figure 2:**
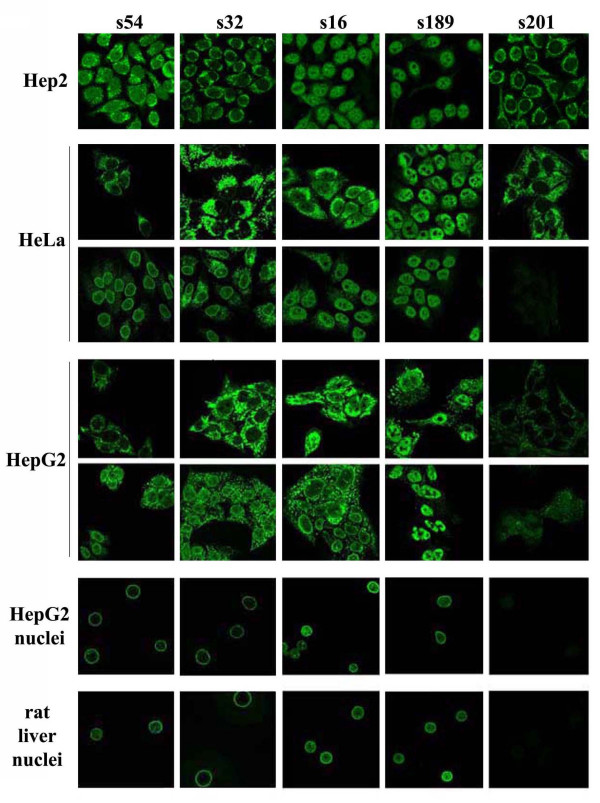
**Validation of substrates used in IIF for the detection of ANEA**. Hep2 slides, HeLa and HepG2 cells fixed with formaldehyde either 4% (upper panel) or 1% (lower panel) and purified nuclei from HepG2 cells and rat liver were incubated with various PBC sera (s54, s32, s16, s189 and s201) and secondary anti-human FITC-labeled IgG antibodies, as described in Materials and Methods.

**Figure 3:**
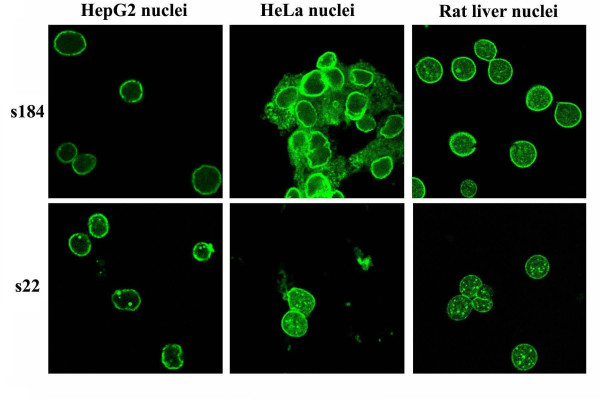
**Purity of isolated nuclei from HEpG2 cells, HeLa cells and rat liver**.

Commercial Hep2-based histology slides, coupled with an anti-IgG-FITC conjugate are routinely used for the detection of circulating autoantibodies. Using this set of reagents, we were able to detect ANEA in 15% of sera. Use of anti-IgM or both anti-IgG and anti-IgM secondary antibodies revealed 13% and 23% of sera positive for ANEA, respectively (Figure 1). However, as we have reported previously [11] the origin and fixation method of cells, used as a substrate, may represent sources of variation of ANEA detection, in patients' sera. In this respect, here we tested HeLa and HepG2 cell lines and isolated nuclei from HepG2 cells and rat liver. The reason for using purified nuclei as substrate for the analysis of autoimmune sera was to eliminate staining from autoantibodies directed against cytoplasmic autoantigens and therefore to enhance sensitivity in the detection of ANEA (Figure 2). We used nuclei isolated from HepG2 cells instead of HeLa cells because unlike nuclei preparations from HeLa cells, those from HepG2 cells were free of cytoplasmic contaminations (Figure 3). We also analyzed sera using nuclei from rat liver because of their high purity after isolation, the convenience of tissue availability and the diversity of normal (no cancerous) cell types that constitute the whole tissue (liver) instead of a cell line. The latter would enhance identification of cell-type specific proteins, because liver contains hepatocytes, Kupffer cells, sinusoidal epithelia, perisinusoidal lipocytes, an endothelial vasculature, and muscle cells.

**Figure 4:**
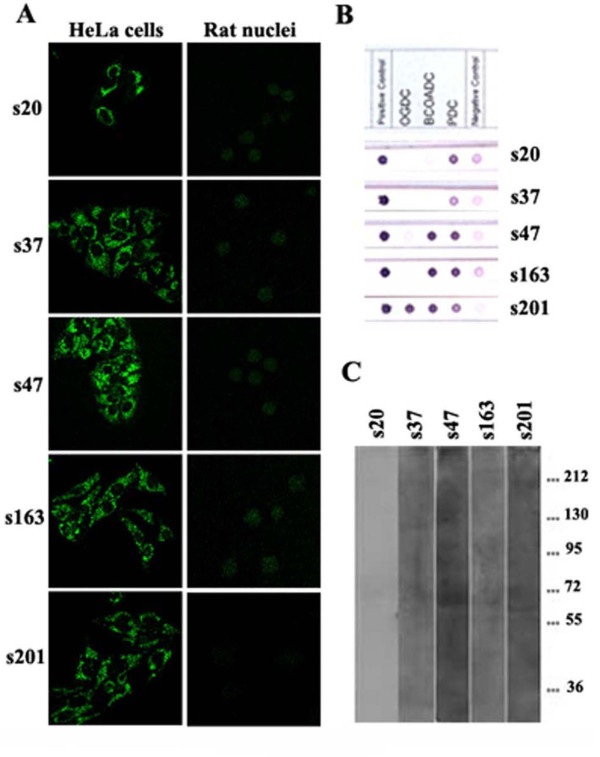
**Purity of nuclear envelopes isolated from rat liver**. (A) Reactivity of PBC sera (s) as detected by IIF on HeLa cells and nuclei isolated from rat liver. Note the cytoplasmic pattern of fluorescence and the absence of staining on nuclei. The same PBC sera were tested (B) for anti-mitochondrial autoantibodies by dot blotting (mitochondria profile blot, Alphadia Diagnostic Products, Wavre, Belgium) and (C) on nuclear envelopes from rat liver by immunoblotting. Note for all sera the presence of anti-mitochondrial autoantibodies and the absence of reactivity on nuclear envelopes.

In Figure 2, we show characteristic patterns observed with a number of patients' sera, which, as will presented below, contain autoantibodies against specific nuclear envelope proteins (anti-gp210-s54, s16, s189-, or anti-lamin A-s32-). The use of different substrates resulted in pattern variability and we showed that, whenever sera contained low titer of ANEA and/or high titer of antibodies directed against cytoplasmic autoantigens, isolated nuclei revealed ANEA more efficiently and easily than whole cells (Figure 2, panels s32, s16 and s189). Interestingly enough, rat liver nuclei, in spite of the species difference, recognize human ANEA with the same efficacy as human liver (HepG2) nuclei. ANEA detection using purified nuclei was specific, as no staining of the nuclear periphery was shown when we used sera containing only cytoplasmic autoantibodies (Figure 2, panel s201, and Figure 4). In addition, we did not detect any fluorescence of the nuclear periphery in both Hep2 commercial slides and purified nuclei when sera from 10 healthy controls, 25 individuals without autoimmune diseases registered with the university hospital, 5 patients with viral hepatitis B, 5 patients with viral hepatitis C and finally 10 patients with alcoholic cirrhosis were analyzed by IIF (not shown).

**Figure 5:**
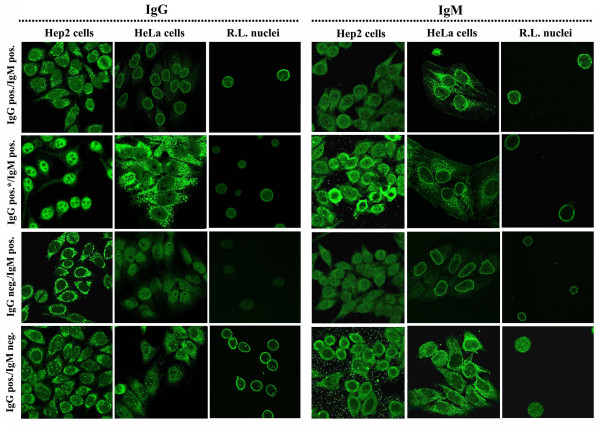
**Detection of ANEA of IgG and IgM class by IIF**. Commercially available slides with Hep2 cells (Hep2 cells), cultured HeLa cells (HeLa cells) and purified nuclei from rat liver (R.L. nuclei) were incubated with various PBC sera and secondary anti-human FITC-labeled IgG and IgM antibodies, as described in Materials and Methods.

Staining on different substrates has been further repeated with an anti-IgM antiserum, to account for isotype-specific ANEA in sera of PBC patients. Representative images are presented in Figure 5. We show that detection of IgG and/or IgM antibodies was depending on the patient's serum, as well as on the substrate used and that isolated nuclei could reveal ANEA more efficiently than whole cells (Figure 5, panel IgG pos.*/IgM pos.).

The prevalence of ANEA as determined in the different substrate/antiserum used varies within the different combinations (Figure 1). Alternative substrates (HeLa cells and isolated nuclei) identify a significantly higher number of sera, positive for ANEA. No significant difference was observed in the prevalence determined using either whole cells or isolated nuclei or when all methods were pooled for positivity in the totality of 103 sera examined. Most sera (44%) had both IgG and IgM antibodies, whereas we found low percentage (4%) of sera containing only IgM antibodies.

In conclusion, these data indicate that substrates and secondary reagents used in immunofluorescence assays are determinant factors for the detection of ANEA. Purified nuclei and appropriately fixed cells are better substrates than commercial Hep2-based histology slides and should be used for the determination of ANEA by IIF when their presence is suspected in patients' sera.

### Correlation of immunofluorescence and immunoblotting in the detection of ANEA

**Figure 6:**
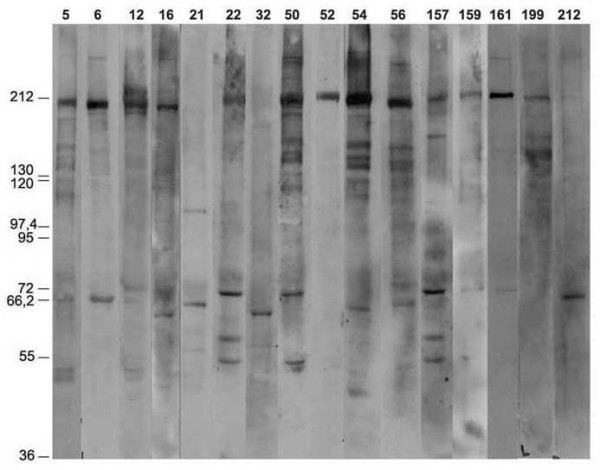
**Detection of ANEA by immunoblotting**. Apparent molecular mass of nuclear envelope proteins from rat liver which are recognized by autoantibodies comprised in selected PBC sera.

In order to confirm our IIF findings, we have tested all sera, by immunoblotting, using purified rat liver nuclear envelopes as substrate. With the exception of sera positive for anti-mitochondrial autoantibodies (Figure 4), all sera positive for ANEA by IIF were also positive by immunoblotting. A variety of reactive protein bands were revealed (Figure 6); most sera bound to proteins with an approximate mass of 210 kDa (35, 34% of sera) and/or 75-55 kDa (39, 37.9% of sera). In order to further characterize the major antigens targeted by circulating autoantibodies, we have analyzed them, using a variety of methods.

### Anti-gp210 autoantibodies prevail in ANEA-positive cases of PBC

**Figure 7:**
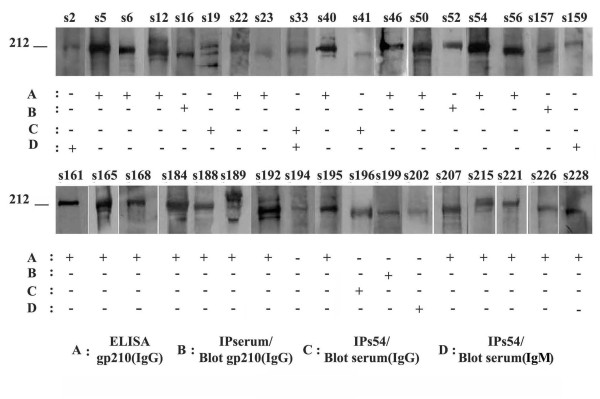
**Prevalence of anti-gp210 autoantibodies**. On the top the reactivity by immunoblotting of the 35 different sera (s) with a protein zone of approximately 210 kDa is shown. The presence (+) or the absence (-) of autoantibodies against gp210 in these sera is assayed by ELISA and immunoprecipitation followed by immunoblotting. For details see text and Figure 8.

Anti-gp210 antibodies were most probable to be present as it is known that these antibodies are PBC-specific. As presented above, our PBC sera revealed autoantibodies for proteins with an apparent molecular mass of ~210 kDa. Using a commercial ELISA kit for anti-gp210 antibody detection, we detected 43.4% of cases positivity, in ANEA IIF-positive sera. However, from the 35 sera, exhibiting autoantibodies against a protein substrate of ~210 kDa, only 23 were identified as anti-gp210 positive with ELISA (Figure 7). This discrepancy could indicate the existence of other, yet unidentified proteins with a similar apparent molecular mass, serving as targets of ANEA autoantibodies. Alternatively, it is also possible that anti-gp210 antibodies are not detected by ELISA because they are directed against epitopes other than the C-terminal of gp210 or belong predominantly to other classes than IgG.

**Figure 8:**
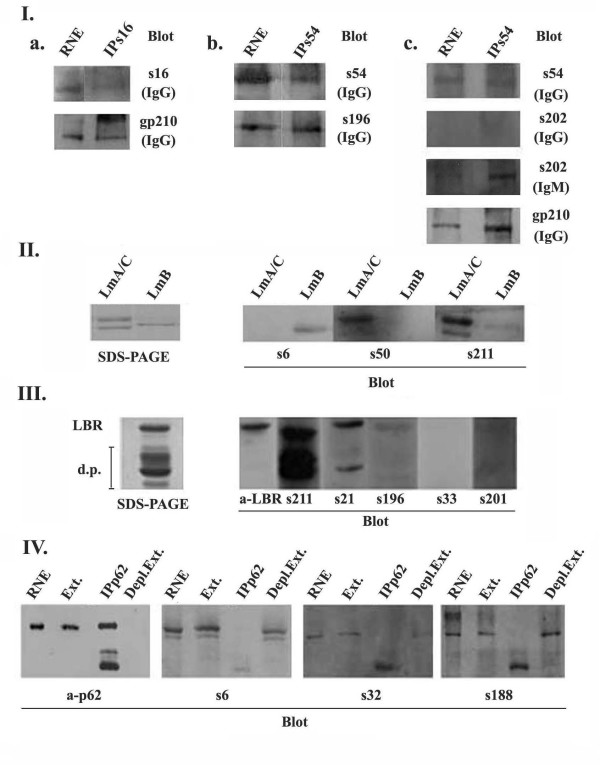
**Identification of specific ANEA by biochemical approaches**. (I) Identification of anti-gp210 autoantibodies. Immunoprecipitation (IP) of gp210 and immunoblotting (Blot) using IgG (IgG) and IgM (IgM) secondary antibodies. (II) Identification of autoantibodies directed against lamins A/C (LmA/C) and B (LmB) by immunoblotting (Blot) using purified proteins (SDS-PAGE). (III) Identification of anti-LBR autoantibodies by immunoblotting (Blot) using purified recombinant LBR (SDS-PAGE). "d.p." denotes characteristic proteolytic products of LBR that are produced after expression in bacteria. Sera negative (s33, s201) and highly (s211, s21) or moderately (s196) positive for LBR are shown. (IV) Identification of anti-p62 autoantibodies.

To discriminate between these possibilities we performed immunoprecipitation experiments using Triton X-100 extracted material from purified rat liver nuclear envelopes. Material precipitated by immunoprecipitation-competent sera was further immunoblotted with anti-gp210 antibodies (Figure 8Ia). Alternatively, for those sera which were not suitable for immunoprecipitation (i.e. s196 and s202), we used serum 54, which was competent for immunoprecipitation and positive for anti-gp210 by ELISA. Immunoprecipitates were then immunoblotted with the different sera to be tested using as detection reagents anti-human IgG and, when necessary, IgM secondary antibodies (Figure 8Ib, c). We found 8/35 additional sera (22.9%) containing anti-gp210 IgG autoantibodies, not revealed by ELISA, whereas 3/35 sera (8.6%) contained anti-gp210 autoantibodies of IgM class alone (Figure 7). The identity of the gp210 substrate was further confirmed by mass spectrometry of the protein immunoprecipitated with sera 16 and 54.

Concluding, our results indicate that 34 of PBC sera (33%) contained anti-gp210 autoantibodies, while 11 of these sera could not be detected by ELISA, necessitating additional assays. Also, there is a statistically significant direct correlation (p < 0.0001) between the 210 kDa zone detected by immunoblotting (35 sera) and the identification of gp210 (34 sera) in the PBC sera tested.

### Prevalence of anti-p62, anti-LBR and anti-lamin autoantibodies in PBC sera

The second major protein band identified in 38% of PBC patients' sera (39 sera), has an apparent molecular mass of 55-75 kDa. Therefore, we investigated the prevalence of anti-p62, anti-LBR and anti-lamin autoantibodies which have been previously reported to occur in autoimmune sera [3].

The presence of anti-p62 autoantibodies was studied by immunoblotting on material immunoprecipitated using specific monoclonal anti-p62 antibodies. As shown in Figure 8IV, monoclonal anti-p62 antibodies (a-p62) recognized p62 nucleoporin in nuclear envelope preparations (RNE), on the material extracted (Ext.) and immunoprecipitated with anti-p62 antibodies (IPp62). Moreover, is shown that there was no detectable p62 in the extract after immunoprecipitation (Depl.Ext.) indicating that almost all of the extracted p62 was absorbed by anti-p62 antibodies. However, none of the patients sera, recognizing an autoantigen of similar molecular mass (55-75 kd) could react with the isolated p62, suggesting the absence of specific anti-p62 autoantibodies. Representative data from selected sera (s6, s32 and s188) are presented in Figure 8IV, where is shown an immunoreactivity for proteins of approximately 60 kDa in the nuclear envelope preparation (RNE) and the extracted material before (Ext.) and after (Depl.Ext.) immunodepletion but not for p62 nucleoporin (IPp62).

**Figure 9:**
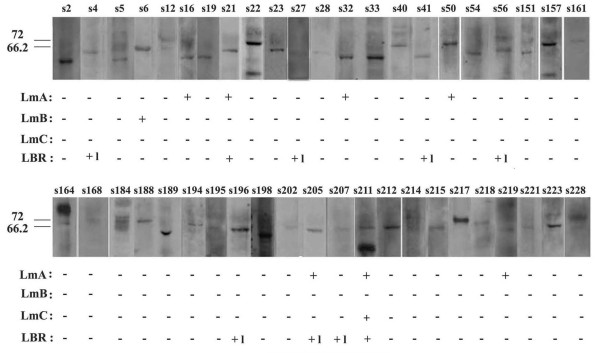
**Prevalence of autoantibodies for lamins and LBR**. On the top the reactivity by immunoblotting of the 39 different sera (s) with a protein zone of molecular mass between 55 and 75 kDa is shown. The presence (+) or the absence (-) of autoantibodies against lamins A, B, C and LBR in these sera is assayed by immunoblotting using purified proteins. "+l" denotes low titer for anti-LBR antibodies.

A similar method was used to investigate the presence of anti-lamin and anti-LBR autoantibodies. They were also detected by immunoblotting on the corresponding purified proteins. Anti-lamin and/or anti-LBR autoantibodies were present in 14 sera; specifically 4 contained anti-lamin A, 1 anti-lamin B, 6 sera contained a low titer anti-LBR antibody, 2 sera contained both anti-lamin A and anti-LBR and finally 1 serum was positive for anti-lamin A/C and anti-LBR antibodies (Figure 9). Figure 8II shows characteristic immunoreactivities for lamin B (s6), lamin A (s50) and both lamins A and C (s211), whereas in Figure 8III are presented profiles from sera without (s33, s201) and with high (s211, s21) or low (s196) titer of anti-LBR autoantibodies. The existence of anti-lamin A autoantibodies was further confirmed by identifying lamin A with mass spectrometry in the immunoprecipitated material using serum 16.

In conclusion, our data indicate the complete absence of anti-p62 autoantibodies, while a small number of sera contained antibodies targeting LBR or nuclear lamins.

## Discussion

Standard immunofluorescence has traditionally a lower sensitivity, as compared to immunoblotting or ELISA, for the detection of autoantibodies; however, molecular-based assays enable the detection of a limited number of selected targets, neglecting others of potential equal or even higher importance. A contribution of the present study is the finding that immunofluorescence may be a powerful technique for the detection of ANEA, which are often sub-optimally detected when autoantibodies of other reactivities are equally present in a specific serum [3,4,11]. In fact, we report high prevalence of autoantibodies against the nuclear periphery (in about half of patients with PBC) when we used multiple substrates (cultured cells and purified nuclei) and antisera (IgG and IgM). In contrast, these antibody classes are under-estimated, when standard IIF procedure (commercially available Hep2 cells and IgG antisera) was used for the detection. Interestingly enough, we further report that heterologous systems (rat liver nuclei/human sera) may also reveal, with a high specificity, ANEA autoantibodies, a technique which might be of potential clinical value.

Previously published studies shows a discrepancy in the results obtained by IIF and immunoblotting or ELISA. For example, in PBC sera, it has been shown that the prevalence of antibodies giving a rim-like membranous fluorescence (10%) by IIF on Hep2 cells was lower than the prevalence of antibodies assayed by ELISA against gp210 (16%), which represent one among other autoantigens of the nuclear envelope. Moreover, for specific sera which were negative for ANEA by IIF, antibodies against gp210 or LBR have been detected using ELISA [9]. Similarly, in another study, anti-NPC antibodies, against at least one of the two major autoantigens at about 200 and 60 kDa, were detected by immunoblotting on purified human NPCs in as many as 22% of the ANA-negative by IIF PBC patients [13]. Our data indicate that an accurate detection of ANEA by IIF can be accomplished if appropriate substrates are used. Cytoplasmic staining, which is a major handicap for the detection of the peripheral nuclear staining very often shown in PBC sera, was considerably decreased or even eliminated when appropriately fixed cells or purified nuclei were used. Sera tested under such conditions showed high prevalence of ANEA by IIF. Of importance, all sera which were positive for anti-gp210 and anti-LBR autoantibodies showed also a peripheral nuclear staining by IIF, indicating that ANEA specific for PBC, are efficiently detected using our experimental protocol.

Another important finding was the identification of undetectable by conventional ELISA, anti-gp210 autoantibodies in more than 10% of PBC sera, representing 21% of sera with ANEA and 32% of anti-gp210 positive sera. Three of 11 sera contained anti-gp210 of only IgM class, whereas anti-gp210 autoantibodies in the last 8 sera did not reacted with the C-terminal epitope using an ELISA commercial kit which is widely used in clinical laboratories. Considering the importance of anti-gp210 antibodies in diagnosis and prognosis of PBC, our results strongly suggest that additional ELISA kits should be developed including the N-terminal epitope as substrate and various secondary reagents for the identification of anti-gp210 autoantibodies of all specificities and Ig class.

Finally, we tried to identify the 60-70 kDa protein zones recognized by almost 40% (39 out of 103 sera) of our sera after immunoblotting on purified rat nuclear envelopes. We identified autoantibodies for LBR or lamins A, B and C, in 14 (36%) of those sera, whereas we have not detected anti-p62 autoantibodies in our PBC sera by immunoprecipitation, although p62 was effectively recognized in the material extracted from rat nuclear envelopes and precipitated using monoclonal anti-p62 antibodies. Moreover, the immunoreactivity of sera with a 60-70 kDa band in nuclear envelope extracts before and after immunoprecipitation clearly indicated that autoantibodies reacting with the 60-70 kDa proteins are not directed against p62 but reacted probably with degradation products of autoantigens of higher M.W. (20 of 25 sera had autoantibodies for gp210) or proteins of the nuclear envelope not yet reported as autoantigens. The only possible explanation for the lack of detection of anti-p62 autoantibodies could be their very low titer. This possibility is supported by the recent identification of autoantibodies against p62 using recombinant rat or human protein in 50% of PBC sera by immunoprecipitation of ^35^S-radioactively labeled p62 and in 40% by immunoblotting [26]. We believe that more studies are needed to confirm the presence and determine the prevalence of anti-p62 antibodies in PBC sera.

## Conclusions

Results of the present study indicate high prevalence of autoantibodies giving rim/peripheral fluorescence in PBC sera when tested by IIF using multiple secondary reagents and adequate substrates for the analysis. We confirm that among many potential ANEA, anti-gp210 antibodies are very specific for PBC, although their sensitivity in PBC sera seems to be underestimated in previous studies. Giving the importance of ANEA and particularly anti-gp210 antibodies in diagnosis and prognosis of PBC, we suggest that modifications emerged by our study, concerning the screening of sera by IIF and the identification of gp210 antibodies, should be included for the identification of ANEA in future studies.

## Competing interests

The authors declare that they have no competing interests.

## Authors' contributions

ET: Acquisition and analysis of data, revising the manuscript. HP: Acquisition of data, technical supervision and revising the manuscript. RS: Technical assistance, preparation of sera and revising the manuscript. MS: Acquisition and analysis of MS data, revising the manuscript. MT: Diagnosis of patients, analysis of data, revising the manuscript. MK: Preparation of sera, follow-up of patients, revising the manuscript. GP: Analysis and interpretation of data, revising the manuscript. EK: Interpretation of data and revising the manuscript. EC: Design of the study, interpretation of data and revising the manuscript. PAT: Conception and design of the study, analysis and interpretation of data, drafting and revising the manuscript. All of the authors read and approved the final manuscript.

## Pre-publication history

The pre-publication history for this paper can be accessed here:

http://www.biomedcentral.com/1471-230X/10/28/prepub

## References

[B1] Georgatos SD Theodoropoulos PA Rules to remodel by: what drives nuclear envelope disassembly and reassembly during mitosis? Crit Rev Eukaryot Gene Expr 1999 9 373 81 10651254 10.1615/critreveukargeneexpr.v9.i3-4.220

[B2] Cronshaw JM Krutchinsky AN Zhang W Chait BT Matunis MJ Proteomic analysis of the mammalian nuclear pore complex J Cell Biol 2002 158 915 27 10.1083/jcb.200206106 12196509 PMC2173148

[B3] Enarson P Rattner JB Ou Y Miyachi K Horigome T Fritzler MJ Autoantigens of the nuclear pore complex J Mol Med 2004 82 423 33 10.1007/s00109-004-0554-z 15175862

[B4] Rigopoulou EI Davies ET Pares A Zachou K Liaskos C Bogdanos DP Rodes J Dalekos GN Vergani D Prevalence and clinical significance of isotype specific antinuclear antibodies in primary biliary cirrhosis Gut 2005 54 528 32 10.1136/gut.2003.036558 15753539 PMC1774444

[B5] Wesierska-Gadek J Penner E Battezzati PM Selmi C Zuin M Hitchman E Worman HJ Gershwin ME Podda M Invernizzi P Correlation of initial autoantibody profile and clinical outcome in primary biliary cirrhosis Hepatology 2006 43 1135 44 10.1002/hep.21172 16628641

[B6] Lassoued K Brenard R Degos F Courvalin JC Andre C Danon F Brouet JC Zine-el-Abidine Y Degott C Zafrani S Antinuclear antibodies directed to a 200-kilodalton polypeptide of the nuclear envelope in primary biliary cirrhosis. A clinical and immunological study of a series of 150 patients with primary biliary cirrhosis Gastroenterology 1990 99 181 6 2188869 10.1016/0016-5085(90)91246-3

[B7] Miyachi K Hankins RW Matsushima H Kikuchi F Inomata T Horigome T Shibata M Onozuka Y Ueno Y Hashimoto E Hayashi N Shibuya A Amaki S Miyakawa H Profile and clinical significance of anti-nuclear envelope antibodies found in patients with primary biliary cirrhosis: a multicenter study J Autoimmun 2003 20 247 54 10.1016/S0896-8411(03)00033-7 12753810

[B8] Miyachi K Shibata M Onozuka Y Kikuchi F Imai N Horigome T Primary biliary cirrhosis sera recognize not only gp210 but also proteins of the p62 complex bearing N-acetylglucosamine residues from rat liver nuclear envelope. Anti-p62 complex antibody in PBC Mol Biol Rep 1996 23 227 34 10.1007/BF00351173 9112233

[B9] Muratori P Muratori L Ferrari R Cassani F Bianchi G Lenzi M Rodrigo L Linares A Fuentes D Bianchi FB Characterization and clinical impact of antinuclear antibodies in primary biliary cirrhosis Am J Gastroenterol 2003 98 431 7 10.1111/j.1572-0241.2003.07257.x 12591064

[B10] Wesierska-Gadek J Hohenuer H Hitchman E Penner E Autoantibodies against nucleoporin p62 constitute a novel marker of primary biliary cirrhosis Gastroenterology 1996 110 840 7 10.1053/gast.1996.v110.pm8608894 8608894

[B11] Tsiakalou V Tsangaridou E Polioudaki H Nifli AP Koulentaki M Akoumianaki T Kouroumalis E Castanas E Theodoropoulos PA Optimized detection of circulating anti-nuclear envelope autoantibodies by immunofluorescence BMC Immunol 2006 7 20 10.1186/1471-2172-7-20 16956395 PMC1574344

[B12] Courvalin JC Lassoued K Bartnik E Blobel G Wozniak RW The 210-kD nuclear envelope polypeptide recognized by human autoantibodies in primary biliary cirrhosis is the major glycoprotein of the nuclear pore J Clin Invest 1990 86 279 85 10.1172/JCI114696 2195063 PMC296718

[B13] Invernizzi P Podda M Battezzati PM Crosignani A Zuin M Hitchman E Maggioni M Meroni PL Penner E Wesierska-Gadek J Autoantibodies against nuclear pore complexes are associated with more active and severe liver disease in primary biliary cirrhosis J Hepatol 2001 34 366 72 10.1016/S0168-8278(00)00040-4 11322196

[B14] Nakamura M Kondo H Mori T Komori A Matsuyama M Ito M Takii Y Koyabu M Yokoyama T Migita K Anti-gp210 and anti-centromere antibodies are different risk factors for the progression of primary biliary cirrhosis Hepatology 2007 45 118 27 10.1002/hep.21472 17187436

[B15] Courvalin JC Lassoued K Worman HJ Blobel G Identification and characterization of autoantibodies against the nuclear envelope lamin B receptor from patients with primary biliary cirrhosis J Exp Med 1990 172 961 7 10.1084/jem.172.3.961 2167346 PMC2188537

[B16] Nickowitz RE Worman HJ Autoantibodies from patients with primary biliary cirrhosis recognize a restricted region within the cytoplasmic tail of nuclear pore membrane glycoprotein Gp210 J Exp Med 1993 178 2237 42 10.1084/jem.178.6.2237 7504063 PMC2191303

[B17] Wesierska-Gadek J Hohenauer H Hitchman E Penner E Autoantibodies from patients with primary biliary cirrhosis preferentially react with the amino-terminal domain of nuclear pore complex glycoprotein gp210 J Exp Med 1995 182 1159 62 10.1084/jem.182.4.1159 7561689 PMC2192276

[B18] Tartakovsky F Worman HJ Detection of Gp210 autoantibodies in primary biliary cirrhosis using a recombinant protein containing the predominant autoepitope Hepatology 1995 21 495 500 7531172

[B19] Bandin O Courvalin JC Poupon R Dubel L Homberg JC Johanet C Specificity and sensitivity of gp210 autoantibodies detected using an enzyme-linked immunosorbent assay and a synthetic polypeptide in the diagnosis of primary biliary cirrhosis Hepatology 1996 23 1020 4 10.1002/hep.510230512 8621127

[B20] Bauer A Habior A Measurement of gp210 autoantibodies in sera of patients with primary biliary cirrhosis J Clin Lab Anal 2007 21 227 31 10.1002/jcla.20170 17621358 PMC6648998

[B21] Bogdanos DP Liaskos C Pares A Norman G Rigopoulou EI Caballeria L Dalekos GN Rodes J Vergani D Anti-gp210 antibody mirrors disease severity in primary biliary cirrhosis Hepatology 2007 45 1583 author reply 1583-4 10.1002/hep.21678 17538935

[B22] Bogdanos DP Pares A Rodes J Vergani D Primary biliary cirrhosis specific antinuclear antibodies in patients from Spain Am J Gastroenterol 2004 99 763 4 author reply 765 10.1111/j.1572-0241.2004.04119.x 15089915

[B23] Gao L Tian X Liu B Zhang F The value of antinuclear antibodies in primary biliary cirrhosis Clin Exp Med 2008 8 9 15 10.1007/s10238-008-0150-6 18385935

[B24] Nakamura M Shimizu-Yoshida Y Takii Y Komori A Yokoyama T Ueki T Daikoku M Yano K Matsumoto T Migita K Yatsuhashi H Ito M Masaki N Adachi H Watanabe Y Nakamura Y Saoshiro T Sodeyama T Koga M Shimoda S Ishibashi H Antibody titer to gp210-C terminal peptide as a clinical parameter for monitoring primary biliary cirrhosis J Hepatol 2005 42 386 92 10.1016/j.jhep.2004.11.016 15710222

[B25] Wesierska-Gadek J Klima A Komina O Ranftler C Invernizzi P Penner E Characterization of autoantibodies against components of the nuclear pore complexes: high frequency of anti-p62 nucleoporin antibodies Ann N Y Acad Sci 2007 1109 519 30 10.1196/annals.1398.058 17785341

[B26] Wesierska-Gadek J Klima A Ranftler C Komina O Hanover J Invernizzi P Penner E Characterization of the antibodies to p62 nucleoporin in primary biliary cirrhosis using human recombinant antigen J Cell Biochem 2008 104 27 37 10.1002/jcb.21595 17960595

[B27] Lin F Noyer CM Ye Q Courvalin JC Worman HJ Autoantibodies from patients with primary biliary cirrhosis recognize a region within the nucleoplasmic domain of inner nuclear membrane protein LBR Hepatology 1996 23 57 61 10.1002/hep.510230109 8550049

[B28] Nickowitz RE Wozniak RW Schaffner F Worman HJ Autoantibodies against integral membrane proteins of the nuclear envelope in patients with primary biliary cirrhosis Gastroenterology 1994 106 193 9 8276182 10.1016/s0016-5085(94)95333-3

[B29] Kaplan MM Primary biliary cirrhosis N Engl J Med 1996 335 1570 80 10.1056/NEJM199611213352107 8900092

[B30] Makatsori D Kourmouli N Polioudaki H Shultz LD McLean K Theodoropoulos PA Singh PB Georgatos SD The inner nuclear membrane protein lamin B receptor forms distinct microdomains and links epigenetically marked chromatin to the nuclear envelope J Biol Chem 2004 279 25567 73 10.1074/jbc.M313606200 15056654

[B31] Blobel G Potter VR Nuclei from rat liver: isolation method that combines purity with high yield Science 1966 154 1662 5 10.1126/science.154.3757.1662 5924199

[B32] Matunis MJ Isolation and fractionation of rat liver nuclear envelopes and nuclear pore complexes Methods 2006 39 277 83 10.1016/j.ymeth.2006.06.003 16870471

[B33] Trinkle-Mulcahy L Boulon S Lam YW Urcia R Boisvert FM Vandermoere F Morrice NA Swift S Rothbauer U Leonhardt H Lamond A Identifying specific protein interaction partners using quantitative mass spectrometry and bead proteomes J Cell Biol 2008 183 223 39 10.1083/jcb.200805092 18936248 PMC2568020

[B34] Mylonis I Chachami G Samiotaki M Panayotou G Paraskeva E Kalousi A Georgatsou E Bonanou S Simos G Identification of MAPK phosphorylation sites and their role in the localization and activity of hypoxia-inducible factor-1alpha J Biol Chem 2006 281 33095 106 10.1074/jbc.M605058200 16954218

